# A frameshift variant in the *SIRPB1* gene confers susceptibility to Crohn’s disease in a Chinese population

**DOI:** 10.3389/fgene.2023.1130529

**Published:** 2023-05-30

**Authors:** Jian Tang, Xingyang Wan, JunXiao Zhang, Na Diao, Caibin Zhang, Xiang Gao, Donglin Ren

**Affiliations:** ^1^Department of Gastroenterology, Guangdong Provincial Key Laboratory of Colorectal and Pelvic Floor Diseases, The Sixth Affiliated Hospital, Sun Yat-sen University, Guangzhou, China; ^2^Department of Colorectal and Anal Surgery, The Sixth Affiliated Hospital, Sun Yat-sen University, Guangzhou, China; ^3^ Institute of Biomedical Sciences, SequMed Biotech Inc., Guangzhou, China; ^4^ Institute of Clinical Pharmacology, School of Pharmaceutical Sciences, Sun Yat-Sen University, Guangzhou, China

**Keywords:** Crohn ‘s disease, han Chinese patients, gene susceptibility, whole gene sequencing, SIRPB1

## Abstract

**Background:** Crohn’s disease (CD), a chronic gastrointestinal inflammatory disease, is increasing in China. With a focus on Han Chinese families with CD, the aim of this study was to find genetic variations that increase CD susceptibility by genome sequencing, genetic association, expression, and functional research.

**Materials and methods:** We performed family-based genome sequencing (WGS) analysis on 24 patients with CD from 12 families and then filtered shared potential causal variants by incorporating association results from meta-analyses of CD GWAS and immunology genes and *in silico* variant effect prediction algorithms. Replication analyses were performed in an independent cohort including 381 patients with CD and 381 control subjects.

**Results:** There were 92 genetic variants significantly associated with CD in Chinese individuals. Among them, 61 candidate loci were validated in replication analyses. As a result, patients carrying a rare frameshift variant (c.1143_1144insG; p. Leu381_Leu382fs) in gene *SIRPB1* had significantly higher risk to develop CD (*p* = 0.03, OR 4.59, 95% CI 0.98–21.36, 81.82% vs. 49.53%). The frameshift variation induced tyrosine phosphorylation of Syk, Akt, and Jak2, elevated the expression of *SIRPB1* at the mRNA and protein levels, activated DAP12, and controlled the activation of NF-κB in macrophages. Additionally, it promoted the synthesis of the pro-inflammatory cytokines IL-1, TNF-, and IL-6.

**Conclusion:** Our results suggest that the rare gain-of-function frameshift variant in *SIRPB1* is associated in Han Chinese patients with CD. The functional mechanism of *SIRPB1* and its downstream inflammatory pathways was preliminarily explored in CD.

## Introduction

Inflammatory bowel disease (IBD), which includes Crohn’s disease (CD) and ulcerative colitis, is a complex polygenic disorder brought on by the improper activation of effector immunologic pathways in those with a genetic predisposition. Genome-wide association studies (GWAS) that look for genetic factors influencing disease start and progression have found 240 IBD-associated loci, which have greatly improved our understanding of the biology underlying these conditions (Anderson et al., 2011; de Lange et al., 2017; de Lange et al., 2017; Franke et al., 2010; Liu et al., 2015). The success of the GWAS approach is supported by the link between neighboring common variations in human populations, but it also makes it challenging to determine with accuracy which variant is causative, the molecular consequences of that variant, and frequently even which gene is perturbed. The association of uncommon protein-altering mutations that are expected to impart a higher risk of disease may help to explain some of the missing heritability in IBD. Because they are linked with fewer neighboring variants, rare variants with greater impact sizes may be easier to understand mechanistically. It is still unknown, nevertheless, how much of the heritability of complex disorders can be attributed to uncommon variations. The promise to better comprehend the molecular and genetic architecture of an exemplary complex disease is thus provided by well-powered investigations of uncommon variations in IBD.

IBD is assumed to have a significant genetic link because the most significant risk factor for the condition at any age is a family history of IBD (Childers et al., 2014; Kuwahara et al., 2012). Patients with IBD who have a family history of the condition frequently present it at a younger age, are more likely to have extra-intestinal manifestations, develop perforating disease, and need longer follow-up than patients without a family history, showing significant an increased genetic susceptibility to the condition (Kuwahara et al., 2012). Therefore, family-based IBD cohort genomic analyses are helpful for understanding the genetic architecture of IBD. IBD susceptibility is influenced by a variety of genetic variables. Whole genome sequencing (WGS), in conjunction with recent significant technological advancements, has made it possible to identify uncommon and novel harmful variants in IBD, providing a deeper understanding of genetic differences within the human genome. Due to this, GWAS have been created to objectively identify genetic risk factors for complex polygenic disorders. According to our hypothesis, rare or novel variations, such as those in genes linked to the innate immune system, are more likely to play a role in the development of CD in patients with a family history of the disease. We used WGS to investigate particular genes or pathways implicated in this disease state. Our capacity to research uncommon variations and ascertain the disease’s genetic origin has been transformed by WGS. To address this question, in this study, we identified and replicated a novel rare causative frameshift variant associated with CD and explored into how it affected functionality.

## Materials and methods

### Study subjects

The genome sequence of 24 Han Chinese patients with CD from 12 families were obtained (Table 1). Written informed consent was provided by the attending parents or legal guardians of the pediatric participants. This study was approved by the Research Ethics Committee (REC) of the Sixth Affiliated Hospital of Sun Yet Sen University (SYSU) (N0.2020ZSLYEC-006).

**TABLE 1:** The clinical characteristics of enrolled families of Crohn’s disease.

Family number	Kinship	Age (years)	Sex	Disease duration (year)	Disease location	Disease behavior	Pelvic disease	Abdominal surgical history
F1-II-1	fraternal twins	18	male	5	L3	B3	yes	yes
F1-II-2		18	male	3	L3	B3	yes	no
F2-II-1	fraternal twins	22	female	7	L3+L4	B1	yes	no
F2-II-2		22	female	8	L3+L4	B1	yes	no
F3-I-3	aunt and nephew	42	female	1	L3	B3	no	yes
F3-II-1		19	male	<1	L3	B1	yes	no
F4-I-2	mother and daughter	45	female	10	L3	B3	yes	yes
F4-II-2		24	female	5	L3	B2	no	no
F5-I-1	father and son	44	male	18	L3	B2	no	yes
F5-II-1		21	male	<1	L3	B1	yes	no
F6-II-1	identical twins	14	female	<1	L3	B2	no	no
F6-II-2		14	Female	<1	L2	B2	yes	no
F7-II-1	elder sister and younger brother	23	female	2	L3	B1	no	no
F7-II-2		21	male	1	L3	B3	no	no
F8-I-1	father and son	44	male	2	L1+L4	B2	yes	no
F8-II-1		20	male	6	L1+L4	B3	yes	no
F9-II-1	elder and younger brother	31	male	1	L3+L4	B1	yes	no
F9-II-2		26	male	2	L3+L4	B1	yes	no
F10-I-1	father and daughter	45	male	7	L1	B2	no	yes
F10-II-1		14	female	5	L3	B3	no	no
F11-II-1	identical twins	37	male	11	L3	B3	no	yes
F11-II-2		37	male	11	L3	B3	no	no
F12-II-1	identical twins	15	female	1	L3	B1	yes	no
F12-II-2		15	female	1	L3	B1	no	no

Montreal classification’ of Crohn’s disease (CD); Disease location (L): L1 terminal ileum, L2 colon, L3 ileocolon, L4 upper gastrointestinal tract; Disease behavior (B): B1 non stricturing non penetrating; B2 stricturing, B3 penetrating.

### WGS

Using a QIAamp DNA Kit (QIAGEN, Hilden, Germany), genomic DNA was extracted from peripheral blood cells in accordance with the manufacturer’s protocol. DNA samples were quantified using a Qubit (Thermo Fisher Scientific). A total of 2 μg of each DNA sample was sent to the Beijing Genome Institute (BGI, Shenzhen, China) for WGS using the BGISEQ-500, according to the manufacturer’s guidelines. According to the manufacturer’s recommendations, the genomic DNA was briefly split by ultrasound on a Covaris E220 (Covaris) to DNA segments between 50 bp—800 bp. The fragmented DNA was then exposed to end-repair, phosphorylation, and A-tailing procedures after being further chosen to 100bp-300bp using AMpure XP Beads (Beckman Coulter, Indiana, United States). The A-tailed segments were ligated to the BGISEQ-500 platform-specific adaptors, and the ligated fragments were then purified and amplified using PCR. Finally, single-stranded DNA circles were produced by the circularization process. The libraries were sequenced using 50 bp paired-end reads on the BGISEQ-500 platform following quantification and qualifying.

### Genome sequence data analysis

SOAPnuke was used to filter the raw sequencing reads (Li et al., 2009b) (N rating >10%, low quality rating >50%, and quality rating<5) and Burrows-Wheeler Aligner (BWA v0.7.17) to align to the UCSC human reference genome (hg19) (Li and Durbin, 2009; Li and Durbin, 2010). The coordinates were sorted using Samtools (version 1.3.1) and duplicates were identified using Picard (version 1.129, http://picard.sourceforge.net) (Li et al., 2009a). Using GATK HaplotypeCaller (McKenna et al., 2010), single nucleotide substitution variants (SNV) and brief insertions and deletions (indels) were identified (McKenna et al., 2010). We used the GATK Variant Quality Score Recalibration (VQSR) that uses machine learning algorithm to filter the raw variant callset. The GATK VQSR used high-quality known variant sets as training and truth resources and built a predictive model to filter spurious variants. The SNPs and InDels marked PASS in the output VCF file were high-confident variation set. For SNPs recalibration strategy, we used the following datasets and features to train the model. (a) Training sets: HapMap V3.3, Omni2.5 M genotyping array data and high-confidence SNP sites produced by the 1000 Genomes Project. (b) Features: Coverage (DP), Quality/depth (QD), Fisher test on strand bias (FS), Odds ratio for strand bias (SOR), Mapping quality rank sum test (MQRankSum), Read position rank sum test (ReadPosRankSum), RMS mapping quality (MQ). For InDels recalibration strategy, we used the following datasets and features to train the model. (a) Training sets: Mills 1000G gold standard InDel set. (b) Features: Coverage (DP), Quality/depth (QD), Fisher test on strand bias (FS), Odds ratio for strand bias (SOR), Mapping quality rank sum test (MQRankSum), Read position rank sum test (ReadPosRankSum).

### Prioritization of variants

All germline SNV and indels were annotated using an in-house annotation pipeline, as described previously (Neveling et al., 2013; de Voer et al., 2013; Vissers et al., 2010). High-confidence calls (i.e., ≥10 reads, ≥5 variant reads, and ≥20% variant reads) were subsequently prioritized for variants that were non-synonymous and were absent in our in-house variant database (2,037 in-house analyzed exomes, mostly from European ancestry). Next, we removed all variants present with a MAF of >0.001 in dbSNPv138, the National Heart, Lung, and Blood Institute (NHLBI) Exome Sequencing Project database (ESP, 6503 exomes, http://evs.gs.washington.edu/EVS/), the Exome Aggregation Consortium (Lek et al., 2016) and Thousand Genome Project. Subsequently, family index patients shared non-synonymous variants that result in alterations in protein function, including protein truncation, splice site defects and missense mutations at highly conserved (phyloP ≥ 3.0) nucleotide positions, were included in our analyses. Alamut v.2.0 software (Interactive Biosoftware) and integrated mutation prediction software (align GVDV, SIFT and PolyPhen-2) (Adzhubei et al., 2010; Kumar et al., 2009; Vissers et al., 2010)packages were used for analyses of the identified variants. The prediction of splicing effects was evaluated based on five different algorithms (SpliceSiteFinder, MaxEntScan, NNSPLICE, GeneSplicer, Human Splicing Finder) through the bioinformatics tools of the Alamut v.2.0 software.

### Identification and selection of variants of candidate genes

By using GWAS, we first targeted germline variations in genes at susceptibility loci known to be linked to IBD (Anderson et al., 2011; de Lange et al., 2017; Ellinghaus et al., 2016; Huang et al., 2017; Julia et al., 2014; Kenny et al., 2012; Liu et al., 2015; Parkes et al., 2007; Yamazaki et al., 2013; Yang et al., 2014). Genetic defects in innate immunity that impair intestinal bacterial sensing are linked to the development of IBD (Cananzi et al., 2021). Recent developments in molecular biology have uncovered crucial details about the genetic basis of numerous inflammatory diseases. Next to the identification of variants in known IBD GWAS genes, we searched for potential pathogenic variants in novel candidate genes using the remaining genome data of our CD family cohort. We concentrated on genes that fulfilled the following criteria while choosing these variants: (Supplementary Table S2): 1) genes with variations that caused protein truncation (such as putative frameshifts, nonsense variations, and variations at canonical splice sites), as well as non-synonymous variations with a PhyloP score of more than 3.0, were chosen; 2) the International Union of Immunological Societies Expert Committee on Primary Immunodeficiency’s collection of primary immune deficiency (PID) genes (Picard et al., 2015); and 3) genes involved in pathways implicated in IBD pathogenesis, including innate immune system, immune system, and neutrophil degranulation pathway (Belinky et al., 2015; Kanegane, 2018).

### Sanger sequencing

After PCR amplification, WGS-identified candidate variant of *SIRPB1* was verified using Sanger sequencing. The Primer3 software program was used to build PCR primers *in silico*. Standard PCR procedures were used on an Applied Biosystems Dual 96-Well GeneAmp PCR System 9,700 (primer sequences available upon request). Using the software package Vector NTI, variant analyses were carried out (Invitrogen, Paisley, United Kingdom).

### Variant validation in independent cohort

Candidate variant validation analysis was performed on 381 probands with IBD and 381 unrelated individuals recruited by the Sixth Affiliated Hospital of SYSU using MassARRAY (Derkach et al., 2013). All individuals recruited were peotected by the REC of the Sixth Affiliated Hospital of SYSU (N0.2020ZSLYEC-006). The phenotypes of the controls were assessed, and no known gastrointestinal or immunological findings were reported. Fisher’s exact testing was conducted, and statistical significance was set at *p* < 0.05 (Derkach et al., 2013).

### Functional validation of the SIRPB1 variant allele

#### Histology

Both historical standard hematoxylin and eosin histological sections from the patient who was found to have the SIRPB1 p. Leu381 Leu382fs variation and IBD controls were assessed.

#### Expression analysis

On 4 μm segments of formalin-fixed paraffin-embedded (FFPE) tissue samples containing terminal ileum tissue from the patient identified as harboring the SIRPB1 p. Leu381 _Leu382fs variation and IBD controls, SIRPB1 expression was analyzed by immunohistochemistry (IHC). Using a BenchMark XT automated tissue staining machine (Ventana Medical Systems, Tucson, AZ, United States), IHC staining was carried out in accordance with the manufacturer’s verified protocols.

### Gene expression data

All the microarray samples used in this study were systematically searched and downloaded from NCBI-GEO (Barrett et al., 2013) after the manual curation of the sample details. We obtained gene expression data of mucosal biopsies in CD patients and normal controls from following array data series: GSE75214, GSE36807 and GSE59071. The bioinformatics online tool GEO2R was used to analyze the mRNA expression of SIRPB1.

### Plasmid transfection

To study the variant type *in vitro* to understand its functional implications in more detail, we established THP-1 cell lines that stably expressed wild-type *SIRPB1* (*SIRPB1* ^*wt*^) and mutant *SIRPB1* (*SIRPB1* ^*11143iG*^, c.1143_1144insG; p. Leu381_Leu382fs). Human *SIRPB1* ^*wt*^ and *SIRPB1* ^*11143iG*^ expression vectors (pEZ-M02/*SIRPB1* ^*wt*^ and pEZ-M02/*SIRPB1* ^*11143iG*^, respectively) were established. According to the manufacturer’s instructions, Lipofectamine 2000 (Invitrogen; Thermo Fisher Scientific, Waltham, MA, United States) was used to transfect THP-1 cells with 0.5 µg of either pEZ-M02/*SIRPB1* ^*wt*^ and pEZ-M02/*SIRPB1* ^*11143iG*^. Continuous neomycin treatment at 450 μg/mL was used to select transfectants that could consistently express the inserted vector plasmid (Thermo Fisher Scientific). The limited dilution approach was used to clone neomycin-resistant cells, which were then kept alive in medium containing neomycin.

### Differentiation of THP-1 cells to macrophages

By administering THP-1 monocytes 100 ng/mL phorbol 12-myristate 13-acetate (PMA; Sigma) for 48 h, the macrophage-like state was generated. Then, THP-1 macrophages were transfected with 0.5 µg pEZ-M02/*SIRPB1* ^*wt*^ and pEZ-M02/*SIRPB1* ^*11143iG*^ plasmid for 4 h. After transfection, THP-1 macrophages were incubated in complete medium for 48 h and stimulated with LPS (100 ng/mL). THP-1 cells were lysed in ice-cold lysis buffer for western blotting, and proteins were separated on 10% SDS page. The main antibodies against DAP12, p-Syk/Syk, p-Akt/Akt, p-Jak2/Jak2, and anti-GAPDH were then used to probe the membranes. According to the manufacturer’s instructions, the culture medium was suspended for ELISA assays and TNF-α, IL-1, and IL-6 ELISA kits were used to detect the substances.

### Statistical analyses

Prism version 8.0 software (GraphPad) was used to perform the statistical analysis. Both the Student’s t-test and the Dunnett’s test were used to determine the significance of differences. When more than two groups were compared, a one-way ANOVA was performed. The data are shown as mean ± SE, and a significance level of 0.05 was used.

## Results

### Clinical characteristics of the enrolled patients with familial CD

**FIGURE 1:**
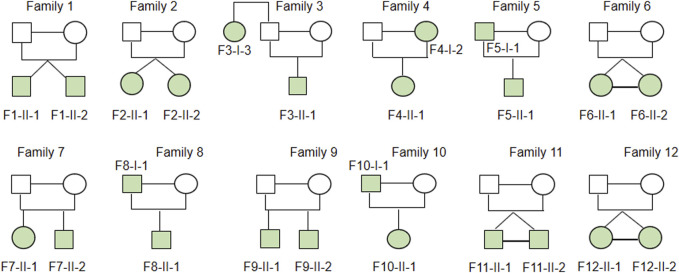
Heritability of enrolled CD family. Two pairs of fraternal twins, three pairs of identical twins, four pairs father or mother and daughter or son, two pairs of elder sister or brother and younger brother, one pair of aunt and niece were included in family based WGS analysis.

Studies of twins and familial clustering of disease clearly indicate that IBD, especially CD, is a hereditary disorder (Halfvarson et al., 2003). Therefore, we hypothesized that family-based CD is more probable to have occurred as a result of uncommon or unique variations and may play a role in the disease’s development. Thus, 24 patients with CD from 12 families in the case database of our IBD center were enrolled for WGS analysis. Table 1 provides a summary of the clinical traits of the individuals that were enrolled. Among them, there were two pairs of fraternal twins, three pairs of identical twins, four pairs of father or mother and daughter or son, two pairs of elder sister or brother and younger brother, and one pair of aunt and niece (Figure 1). All the patients were of Han origin, including 13 men (54.17%) and 11 women (45.83%). The median age of participants in this study was 22 years (range 14–45 years). Regarding disease location, 20 patients were diagnosed with ileocolonic inflammation (L3, 83.33%), one patient with colonic inflammation (L2, 4.17%), 3 patients with ileal inflammation (L1, 12.50%), and six patients had concomitant upper gastrointestinal disease (25.00%). Regarding disease behavior, nine patients were accompanied with non-stricturing non-penetrating lesions (B1, 37.50%), six patients had stricturing lesions (B2, 25.00%), and nine patients had penetrating lesions (B3, 37.50%). A total of 13 patients (54.17%) had pelvic disease and six patients (25.00%) had a history of abdominal surgery.

### IBD susceptibility analysis of identified gene variant

To further understand the genetic basis of IBD, we used WGS to look for uncommon, possibly disease-causing coding variations in familiar CD patients (Table1). A substantial percentage of coding variants discovered across individuals in a family were prioritized for further exploration using a series of progressive variant filters. We identified on average 3,430,329 variants (range: 3,393,972–3,466,204) per genome. A prioritization scheme was applied to identify candidate variants as shown in table 2. After rigorous bioinformatics analysis steps (see Methods), there were 92 genetic variants significantly associated with CD in Chinese individuals including several known IBD related gene including *NOD2, ZPBP2, TNFSF15, LSP1, SDCCAG3, MUC19*. Then, we adopted the MassArray Analyzer system (Sequenom, Inc., SanDiego, CA, United States) for genotyping those genetic variants. We designed primers for 89 variants in Sequenom official websites, while, there is suitable primer for the remaining 3 variants. However, those 89 primers were distributed in 5 panels, due to the limited budget, only the top 2 largest panels (a total of 61 variants were included, about 30 variants per panel) used for the subsequently assay. We finally generated a candidate variants list including 61 variants for replication analyses with an independent cohort including 381 patients with CD and 381 control subjects (Table 3).

**TABLE 2:** Prioritization scheme for genome data analysis of 24 CD patients.

Type of prioritization filter	Remaining variants (*n*)
All variants	82,327,899
Coding region and canonical splice site variants after quality filtering (total ≥ 10 reads, ≥ 5 variant reads and ≥ 20% variant reads)	13,819
Non-synonymous variants, canonical splice site variants	9,833
Family index patients sharedVariants that result in alterations in protein function (protein truncation, splice site defects and missense mutations at highly conserved (phyloP ≥ 3.0) nucleotide positions.	2,749
Not in in-house database and MAF ≤ 0.001 in dbSNPv138, ESP, ExAC and Thousand Genome Project	2,007
Variants in genes at susceptibility loci known to be linked to IBD, primary immune deficiencies gene and IBD-related pathway genes and novel candidate variants	132
Variants pass IGV check	92
Variants for validation in large cohort	61 (4 varints in GWAS genes, 22 variants in IBD related pathway genes and 35 novel variants)

**TABLE 3:** IBD susceptibility analysis of identified gene variants.

Gene	r*s number*	Unique ID	Genotype (mutation vs. wild type)	Genotype frequency (mutation carries vs. wild type carries)	P value ^a^	OR	95% CI
*PCNXL2*	rs759917992	chr1:233386580T>C	TT^b^	NA^c^	NA^c^	NA^c^	NA^c^
*TP73*	rs1641123267	chr1:3638706C>T	CT vs. CC^d^	**Patients group:**	1.000	1.00	1.00-1.01
				1/379(0.26%) vs. 378/379(99.74%)			
				**Control group:**			
				0/365(0.00%) vs. 365/365(100.00%)			
*UBE2U*	rs776567811	chr1:64676467AC>A	AC^b^	NA^c^	NA^c^	NA^c^	NA^c^
*LSP1*	NA	chr11:1891892G>A	CC^b^	NA^c^	NA^c^	NA^c^	NA^c^
*C11orf42*	rs751350529	chr11:6231170C>T	CT vs. CC^d^	**Patients group:**	0.499	1.00	0.99-1.00
				0/379(0.00%) vs. 379/379(100.00%)			
				**Control group:**			
				2/380(0.53%) vs. 378/380(99.47%)			
*C11orf42*	rs749213397	chr11:6231682G>A	GA vs. GG^d^	**Patients group:**	0.122	0.99	0.98-1.00
				0/380(0.00%) vs. 380/380(100.00%)			
				**Control group:**			
				3/374(0.80%) vs. 371/374(99.20%)			
*ALG8*	rs1488580557	chr11:77813960G>A	GG^b^	NA^c^	NA^c^	NA^c^	NA^c^
*POLR3B*	rs2036678883	chr12:106770153C>T	CC^b^	NA^c^	NA^c^	NA^c^	NA^c^
*OAS3*	rs771052891	chr12:113385843GC>G	GC.G vs. GC.GC^d^	**Patients group:**	0.287	2.95	0.59-14.72
				6/380(1.58%) vs. 374/380(98.42%)			
				**Control group:**			
				2/370(0.54%) vs. 368/370(99.46%)			
*MUC19*	rs1411441474	chr12:40834995T>A	TA vs. TT^d^	**Patients group:**	0.173	0.32	0.07-1.61
				2/380(0.79%) vs. 378/380(99.47%)			
				**Control group:**			
				6/372(1.61%) vs. 366/372(98.39%)			
*MYO1A*	NA	chr12:57440644TAC>T	TAC.TAC^b^	NA^c^	NA^c^	NA^c^	NA^c^
*A2ML1*	rs766100204	chr12:8998099G>A	GA vs. GG^d^	**Patients group:**	1.000	1.95	0.18-21.56
				2/374(0.53%) vs. 372/374(99.47%)			
				**Control group:**			
				1/363(0.28%) vs. 362/363(99.72%)			
*EPSTI1*	NA	chr13:43537471T>TA	TT^b^	NA^c^	NA^c^	NA^c^	NA^c^
*DAAM1*	NA	chr14:59782026G>T	GT vs. GG^d^	**Patients group:**	1.000	1.00	1.00-1.01
				1/378(0.26%) vs. 377/378(99.74%)			
				**Control group:**			
				0/364(0.00%) vs. 364/364(100.00%)			
*SYNE2*	rs777169796	chr14:64519635A>T	AT vs. AA^d^	**Patients group:**	1.000	1.00	1.00-1.01
				1/378(0.26%) vs. 377/378(99.74%)			
				**Control group:**			
				0/354(0.00%) vs. 354/354(100.00%)			
*CPPED1*	rs748886359	chr16:12798820C>T	CC^b^	NA^c^	NA^c^	NA^c^	NA^c^
*BEAN1*	rs989514270	chr16:66471600C>A	CC^b^	NA^c^	NA^c^	NA^c^	NA^c^
*AATF*	rs1306922955	chr17:35307665G>A	GG^b^	NA^c^	NA^c^	NA^c^	NA^c^
*ZPBP2*	rs1460554471	chr17:38024800A>G	AA^b^	NA^c^	NA^c^	NA^c^	NA^c^
*PDK2*	rs748085033	chr17:48185985C>T	CT + TT vs. CC	**Patients group:**	1.000	1.01	0.06-16.13
				1/380(0.26%) vs. 379/380(99.74%)			
				**Control group:**			
				1/382(0.26%) vs. 381/382(99.74%)			
*ENO3*	rs764120380	chr17:4856098C>T	CC^b^	NA^c^	NA^c^	NA^c^	NA^c^
*EPX*	rs757233476	chr17:56281773C>T	CT vs. CC^d^	**Patients group:**	0.499	1.00	1.00-1.01
				1/379(0.26%) vs. 378/379(99.74%)			
				**Control group:**			
				0/381(0.00%) vs. 381/381(100.00%)			
*COL5A3*	NA	chr19:10079057	TCACAGGGTCTCC.T vs. TCACAGGGTCTCC.TCACAGGGTCTCC^d^	**Patients group:**	0.499	1.00	1.00-1.01
				1/379(0.26%) vs. 378/379(99.74%)			
		TCACAGGGTCTCC>T		**Control group:**			
				0/381(0.00%) vs. 381/381(100.00%)			
***TYK2***	NA	**chr19:10479075**	**GAAGC.G + GG** vs. **GAAGC.GAAGC**	**Patients group:**	**0.006**	**0.10**	**0.01-0.76**
				**1/380**(**0.26%**) vs. 379/380(99.74%)			
		**GAAGC>G**		**Control group:**			
				**10/375**(**2.67%**) vs. 365/375(97.33%)			
*GDF1*	rs1568291627	chr19:18981025	ACGGGGGCG.A + AA vs. ACG​GGG​GCG.ACG​GGG​GCG	**Patients group:**	1.000	0.96	0.06-15.33
				1/381(0.26%) vs. 380/381(99.74%)			
		ACGGGGGCG>A		**Control group:**			
				1/364(0.27%) vs. 363/364(99.73%)			
*C19orf40*	rs760353712	chr19:33464372C>CTT	C. CTT vs. CC^d^	**Patients group:**	0.499	1.00	1.00-1.01
				1/377(0.27%) vs. 376/377(99.73%)			
				**Control group:**			
				0/378(0.00%) vs. 378/373787(100.00%)			
*VRK3*	rs1048569809	chr19:50528523C>G	CG vs. CC^d^	**Patients group:**	0.373	4.03	0.45-36.24
				4/380(1.05%) vs. 376/380(98.95%)			
				**Control group:**			
				1/380(0.26%) vs. 379/380(99.74%)			
*DFNB59*	rs1437628682	chr2:179320735A>G	AG vs. AA^d^	**Patients group:**	0.394	1.02	0.99-1.05
				1/67(1.49%) vs. 66/67(98.51%)			
				**Control group:**			
				0/103(0.00%) vs. 103/103(100.00%)			
*VRK2*	rs1328945383	chr2:58312086G>A	GG^b^	NA^c^	NA^c^	NA^c^	NA^c^
*WDPCP*	NA	chr2:63486522T>TC	T.TC vs. TT^d^	**Patients group:**	1.000	1.00	1.00-1.01
				1/380(0.26%) vs. 379/380(99.74%)			
				**Control group:**			
				0/345(0.00%) vs. 345/345(100.00%)			
*ANKEF1*	rs752349062	chr20:10019057C>A	CC^b^	NA^c^	NA^c^	NA^c^	NA^c^
*SIRPB1*	rs1275744950	chr20:1546854GC>GCC	GC.GCC vs. GC^d^	**Patients group:**	**0.034**	**4.59**	**0.98-21.36**
				9/381(2.36%) vs. 372/381(97.64%)			
				**Control group:**			
				2/381(0.52%) vs. 379/381(99.48%)			
*CPNE1*	NA	chr20:34214629C>CT	C.CT vs. CC^d^	**Patients group:**	1.000	1.00	1.00-1.01
				1/380(0.26%) vs. 379/380(99.74%)			
				**Control group:**			
				0/380(0.00%) vs. 380/380(100.00%)			
*PLCG1*	NA	chr20:39792446A>T	AA^b^	NA^c^	NA^c^	NA^c^	NA^c^
*FBXO40*	NA	chr3:121341344CT>C	CT.C vs. CT.CT^d^	**Patients group:**	1.000	1.00	1.00-1.01
				1/379(0.26%) vs. 378/379(99.74%)			
				**Control group:**			
				0/379(0.00%) vs. 379/379(100.00%)			
*ERC2*	rs1293707325	chr3:56183136G>A	GG^b^	NA^c^	NA^c^	NA^c^	NA^c^
*MMAA*	rs757548934	chr4:146572222C>T	CT vs. CC^d^	**Patients group:**	1.000	1.00	1.00-1.01
				1/380(0.26%) vs. 379/380(99.74%)			
				**Control group:**			
				0/339(0.00%) vs. 339/339(100.00%)			
*KLHL5*	rs755006031	chr4:39105132G>GT	GG^b^	NA^c^	NA^c^	NA^c^	NA^c^
*SEC31A*	NA	chr4:83788384G>A	GG^b^	NA^c^	NA^c^	NA^c^	NA^c^
*SLC2A9*	NA	chr4:9922067C>T	CT vs. CC^d^	**Patients group:**	1.000	1.00	1.00-1.00
				0/380(0.00%) vs. 380/380(100.00%)			
				**Control group:**			
				1/382(0.26%) vs. 381/382(99.74%)			
*FBXL21P*	rs201662172	chr5:135273232C>A	CA vs. CC^d^	**Patients group:**	1.000	1.00	1.00-1.01
				1/379(0.26%) vs. 378/379(99.74%)			
				**Control group:**			
				0/379(0.00%) vs. 379/379(100.00%)			
***PCDH12***	NA	**chr5:141336148C>T**	**CT vs. CC** ^d^	**Patients group:**	**0.006**	**0.10**	**0.01-0.77**
				1/380(0.26%) vs. 379/380(99.74%)			
				**Control group:**			
				10/380(2.63%) vs. 370/380(97.37%)			
*GHR*	rs752025877	chr5:42565977A>G	AA^b^	NA^c^	NA^c^	NA^c^	NA^c^
*SYNJ2*	NA	chr6:158438246	AAAGG^b^	NA^c^	NA^c^	NA^c^	NA^c^
		AAAGG>A					
***CAGE1***	rs1414911763	**chr6:7329418G>A**	**GA vs. GG** ^d^	**Patients group:**	**1.9** × **10** ^**-5**^	**0.90**	**0.86-0.94**
				0/174(0.00%) vs. 174/174(100.00%)			
				**Control group:**			
				23/232(9.91%) vs. 209/232(90.09%)			
*SH2B2*	NA	chr7:101960938C>T	CC^b^	NA^c^	NA^c^	NA^c^	NA^c^
*RPA3*	rs529874466	chr7:7758145C>G	CG vs. CC^d^	**Patients group:**	1.000	0.84	0.05-13.55
				1/373(0.27%) vs. 372/373(99.73%)			
				**Control group:**			
				1/315(0.32%) vs. 314/315(99.68%)			
*CD36*	rs748202229	chr7:80285946C>T	CC^b^	NA^c^	NA^c^	NA^c^	NA^c^
*TNFSF15*	NA	chr9:117568285T>C	TC + CC vs. TT	**Patients group:**	0.499	1.01	1.00-1.01
				2/381(0.52%) vs. 379/381(99.48%)			
				**Control group:**			
				0/381(0.00%) vs. 381/381(100.00%)			
*LRSAM1*	rs770106776	chr9:130265135G>A	GA + AA vs. GG	**Patients group:**	0.622	0.49	0.04-5.44
				1/379(0.26%) vs. 378/379(99.74%)			
				**Control group:**			
				2/373(0.54%) vs. 371/373(99.46%)			
*HMCN2*	rs1445146226	chr9:133245203G>A	GA vs. GG^d^	**Patients group:**	0.499	1.00	1.00-1.01
				1/379(0.26%) vs. 378/379(99.74%)			
				**Control group:**			
				0/381(0.00%) vs. 381/381(100.00%)			
*SDCCAG3*	rs375609278	chr9:139301649C>G	CG vs. CC^d^	**Patients group:**	0.490	1.00	0.99-1.00
				0/380(0.00%) vs. 380/380(100.00%)			
				**Control group:**			
				1/365(0.27%) vs. 364/365(99.73%)			
*PRSS3*	NA	chr9:33794797TGA>T	TGA^b^	NA^c^	NA^c^	NA^c^	NA^c^
*CCL27*	rs746707552	chr9:34662369G>A	GG^b^	NA^c^	NA^c^	NA^c^	NA^c^
*PCSK5*	rs769457551	chr9:78973443C>T	CT vs. CC^d^	**Patients group:**	1.000	1.00	0.06-16.05
				1/380(0.26%) vs. 379/380(99.74%)			
				**Control group:**			
				1/380(0.26%) vs. 379/380(99.74%)			
*NOD2*	rs104895438	chr16:50745656G>A	GG^b^	NA^c^	NA^c^	NA^c^	NA^c^
***MUC19***	**rs112524759**	**chr12:40882387TA>T**	**TA.T + TT vs. TA.TA**	**Patients group:**	**0.03**	**1.01**	**0.73-1.40**
				97/379(25.59%) vs. 282/379(74.41%)			
				**Control group:**			
				94/370(25.41%) vs. 276/370(74.59%)			
*BIRC8*	rs145690856	chr19:53793456G>A	GG^b^	NA^c^	NA^c^	NA^c^	NA^c^
*XPA*	rs149226993	chr9:100447247G>A	GG^b^	NA^c^	NA^c^	NA^c^	NA^c^
*GLB1*	rs192732174	chr3:33109737G>A	GG^b^	NA^c^	NA^c^	NA^c^	NA^c^
*ERCC4*	rs2020959	chr16:14041622C>A	CA vs. CC^d^	**Patients group:**	1.00	1.00	1.00-1.01
				1/377(0.27%) vs. 376/377(99.73%)			
				**Control group:**			
				0/363(0.00%) vs. 363/363(100.00%)			

^a^ Chi-Square Tests or Fisher's Exact Test;

^b^ Only one genotype was detected;

^c^ Not available, because this mutation/SNP have no minor allele;

^d^ Only two genotypes were detected; These P-values ≤0.05 were highlighted in bold font.

### Gain-of-function frameshift variant SIRPB1 p. Leu381_Leu382fs in a cohort of patients with CD and healthy controls

**FIGURE 2:**
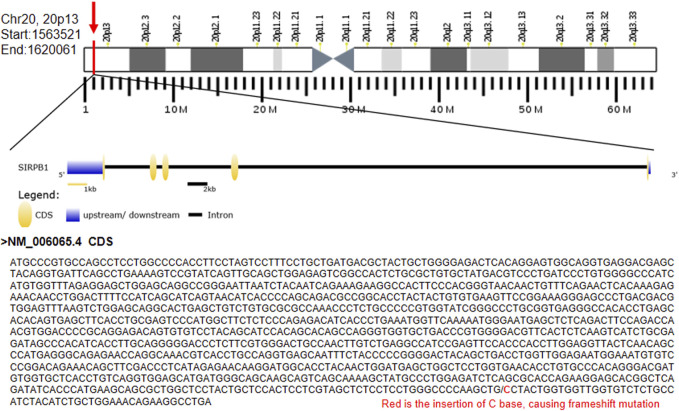
Identification of a rare frameshift mutation of *SIRPB1* gene A. rare frameshift mutation (c.1143_1144insG; p. Leu381_Leu382fs) was identified in an innate immune gene SIRPB1 in both probands of Family CD-7.

Notably, we identified the frameshift variant *SIRPB1* p. Leu381_Leu382fs in two index patients, F7-II-1 and F7-II-2, from Family 7 (Figure 2). Patient F7-II-1 was the elder sister of patient F7-II-2, whose age was 23 years with a disease duration of about 2 years, and patient F7-II-2 was the younger brother whose age was 21 years with a disease duration of approximately 1 year. Both manifested as ileocolonic inflammation (L3). The disease behavior of F7-II-1 was non-stricturing non-penetrating (B1), whereas the disease behavior of F7-II-2 was penetrating (B3). For replication analysis, the blood sample of 384 CD patients including 104 females and 280 males in our database were used for microarray analysis to verify the susceptibility gene mutation. The mean age of these patients was 27.0 ± 10.4 years-old. The 384 blood samples of control group were collected from healthy testing population including 170 female and 214 female and the mean age of these patients was 34.7 ± 9.1 years-old. The unique uncommon frameshift variant in *SIRPB1* was considerably enriched in CD patients compared to controls, according to genotyping analysis (*p* = 0.03, OR 4.59, 95% CI 0.98–21.36, 81.82% vs. 49.53%). (Table 3). The clinical characteristics of patients with CD identified to harbor the SIRPB1 p. Leu381_Leu382fs variant are listed in Table 4.

**TABLE 4:** The clinical characteristics of CD patients with *SIRPB1* gene mutation.

Patient number	Age (years)	sex	Disease duration (year)	Disease location	Disease behaviour	Pelvic disease	Abdominal surgical history
1	23	female	2	L3	B1	yes	no
2	21	male	1	L3	B3	no	no
3	20	male	<1	L3	B1	no	no
4	17	male	1	L3	B1	yes	no
5	42	male	8	L3	B3	no	yes
6	15	female	5	L3	B1	yes	no
7	31	male	6	L3	B3	no	yes
8	21	male	2	L3	B1	no	no
9	54	male	20	L1	B1	no	no

Montreal classification’ of Crohn’s disease (CD); Disease location (L): L1 terminal ileum, L2 colon, L3 ileocolon, L4 upper gastrointestinal tract; Disease behavior (B): B1 non stricturing non penetrating; B2 stricturing, B3 penetrating.

### IHC analysis of the SIRPB1 p. Leu381_Leu382fs variant

**FIGURE 3:**
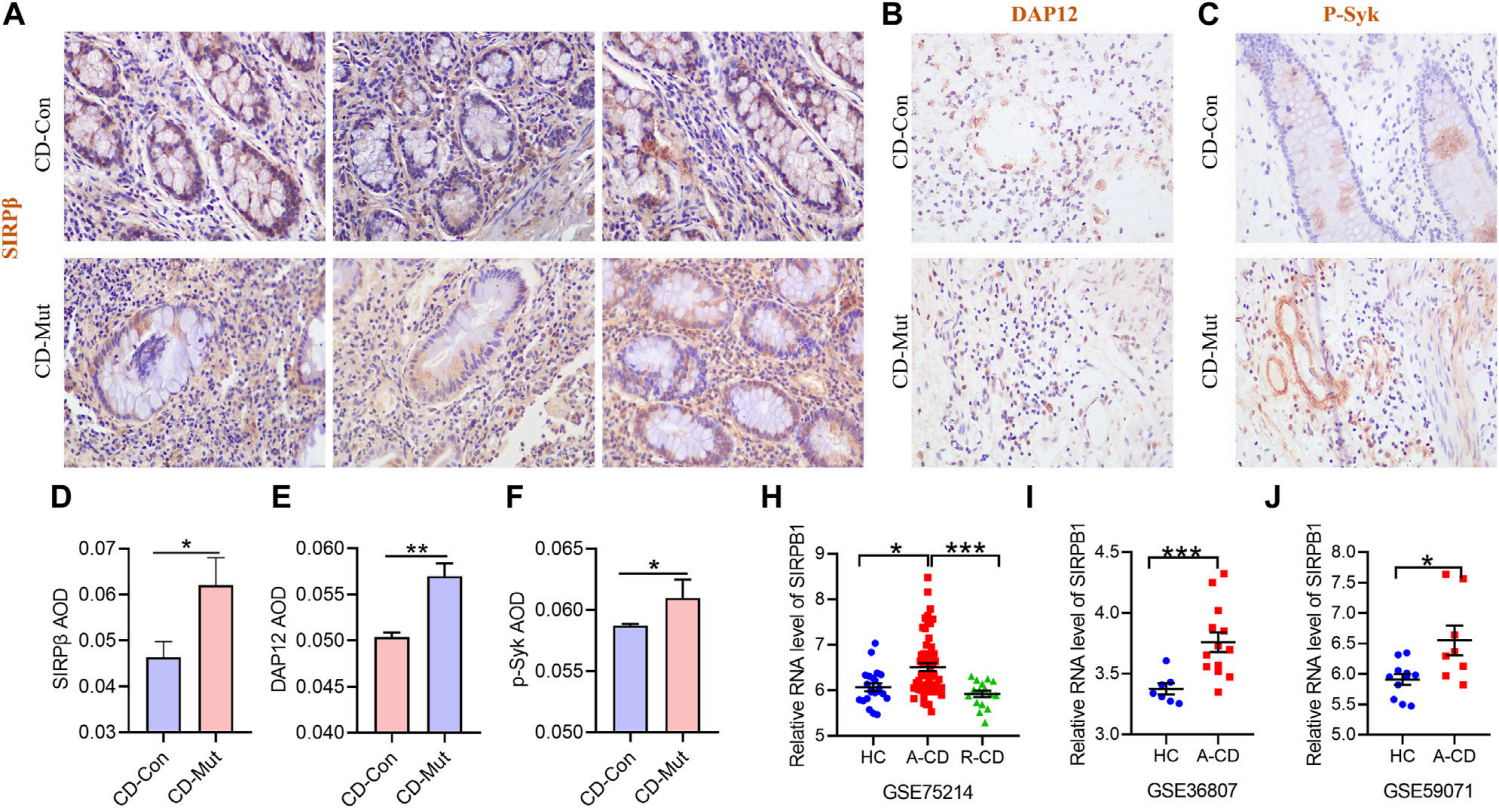
Higher expression of *SIRPB1* in mutant CD patients. A/B/C Immunohistochemistry (IHC) staining of SIRPβ **(A)**, DAP12 **(B)**, and p-Syk **(C)** in ileocolonic tissue from wild type CD patients and control patients; **(D,E,F)** Statistical analyses analysis of IHC staining; G/H/I *SIRPB1* expression was assessed using a publicly accessible data. The following array data series were analyzed to generate the human patient expression data: GSE75214; GSE36807; GSE59071. GSE75214 **(G)**: mucosal biopsies of ileal mucosa of 11 health controls (HC), 15 inactive CD patients (R-CD) and 50 active CD patients (A-CD) were used to analyze mRNA expression of SIRPB1. GSE36807 **(H)**: intestinal biopsies from 7 health controls (HC), and 15 CD patients were used to analyze mRNA expression of SIRPB1. GSE59071 **(I)** mucosal biopsies were obtained at endoscopy from 8 health controls (HC) and Crohn’s disease (CD) patients.

A member of the family of signal-regulating proteins (SIRP) and of the immunoglobulin superfamily, SIRPβ (also known as CD172b) is encoded by *SIRPB1* (van den Berg et al., 2005). Previous research on *SIRPB1* mostly focused on its biochemical properties and functions and discovered that it stimulates DAP12 and Syk tyrosine phosphorylation, which then activates the mitogen-activated protein kinase (MAPK) pathway to increase phagocytosis in macrophages (Hayashi et al., 2004). However, the role of *SIRPB1* in IBD pathogenesis has not yet been reported. Therefore, we first assessed the expression levels of *SIRPB1* by analyzing publicly available data from patients with IBD and healthy individuals. According to the findings, patients with active CD (A-CD) had considerably higher levels of *SIRPB1* expression in their ileocolonic tissue than either healthy individuals or patients with CD that was in remission (R-CD) (Figures 3G–I), indicating that *SIRPB1* is possibly involved in the progression of CD. Next, we assessed the expression levels of SIRPβ in the ileocolonic tissue of variant and wild-type patients with CD using IHC (Supplementary Table S3). Notably, patients with CD and the *SIRPB1* variant exhibited significantly higher levels of SIRPβ than those with CD and wild-type *SIRPB1* (Figures 3A,D). Consistently, SIRPβ-mediated transduction signal molecules, such as DAP12 and p-Syk, were also significantly upregulated in patients with CD and the variant compared with WT controls (Figures 3B,C,E,F). Our data suggest that the higher expression of SIRPβ was caused by the frameshift variation of *SIRPB1*.

### Functional characterization of the rare SIRPB1 frameshift variant (c.1143_1144insG; p. Leu381_Leu382fs)

**FIGURE 4:**
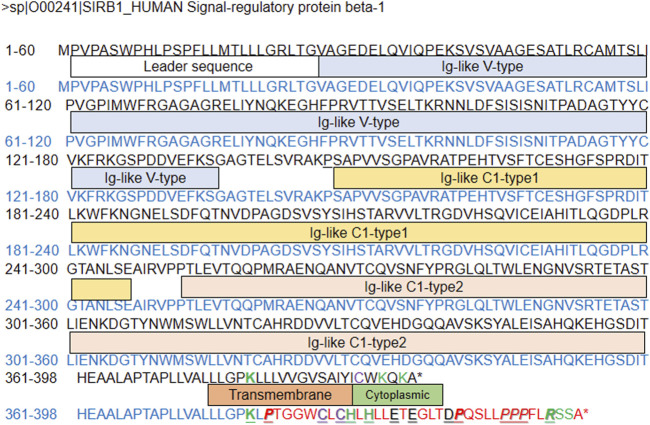
Predicted amino-acid sequence from wild-type control and patients with frameshift SIRPB1 mutation.

As documented above, the frameshift variant in *SIRPB1* led to higher expression of SIRPβ; however, the function of the rare *SIRPB1* frameshift variant still needs to be elucidated. The interaction of SIRPβ with the activating adaptor protein DAP12, which carries an immunoreceptor tyrosine-based activation motif (ITAM) and transmits activating signals, depends on the presence of a basic amino acid side chain in the transmembrane domain of SIRPβ (Lanier and Bakker, 2000; Tomasello and Vivier, 2005). An earlier study showed that phosphorylating Syk and MAPK and crosslinking mouse SIRPβ with monoclonal antibodies increases neutrophil migration and macrophage phagocytosis (Hayashi et al., 2004; Liu et al., 2005). As shown in Figure 4, the frameshift variant of *SIRPB1* gene led to an alteration of the amino acid sequence located both on the transmembrane and the cytoplasm. Therefore, we hypothesized that the frameshift variant of *SIRPB1* could make functional contributions to the inflammation process.

**FIGURE 5:**
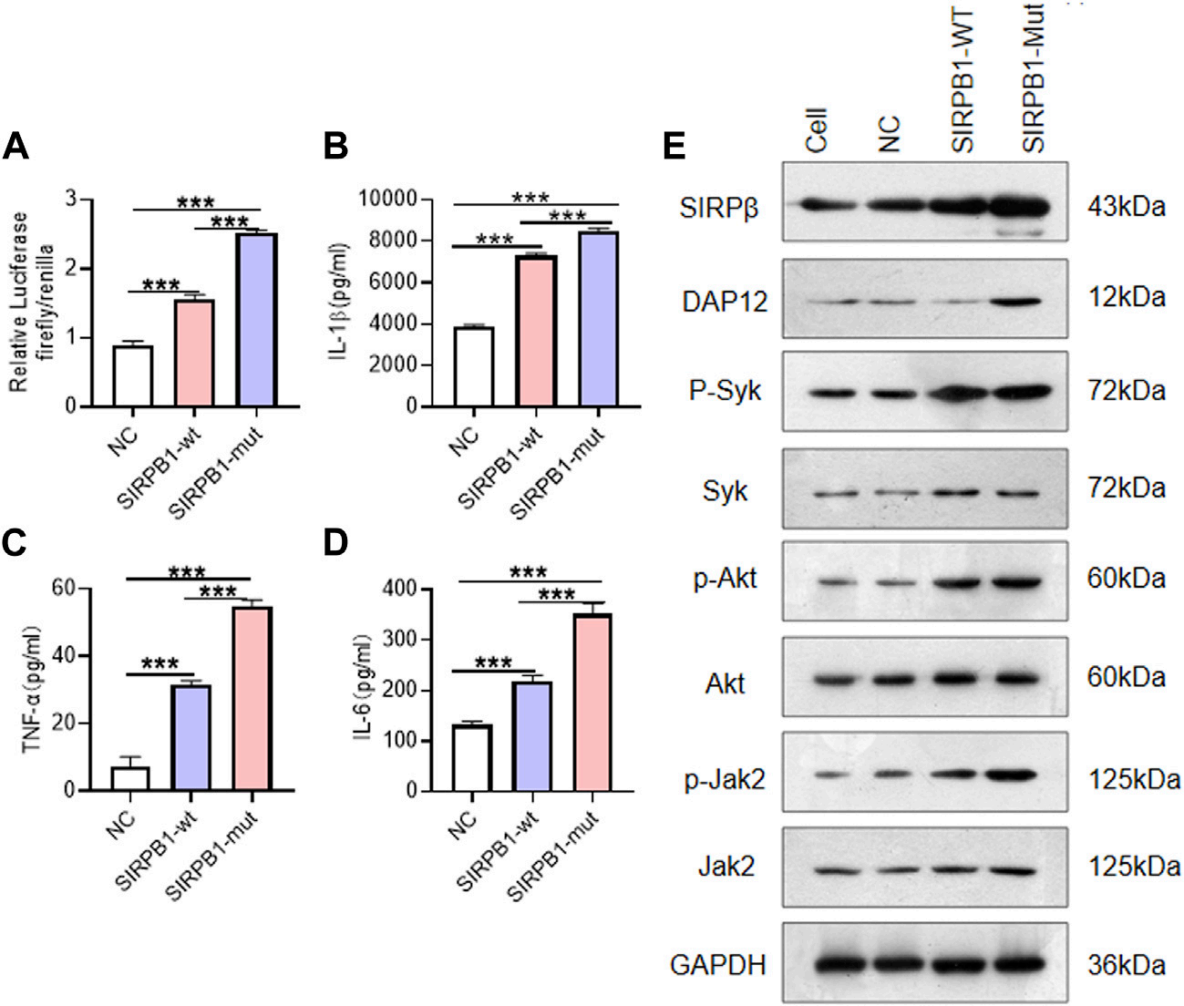
SIRPB1 mutation promote inflammatory response of macrophages *in vitro*. **(A)** NF-kB transcriptional activity of THP-1 cells from different groups was detected by a luciferase reporter assay; **(B,C,D)** IL-1β, IL-6 and TNFα levels in culture medium of THP-1 cells from different groups; **(E)** Western Blotting of signal transduction molecules in THP-1 cells from different groups.

To investigate the potential effects of the identified novel frameshift variant of *SIRPB1*, Plasmids carrying either *SIRPB1* ^*wt*^ and *SIRPB1* ^*11143iG*^ sequence were transfected into THP-1 cells that had been stimulated with PMA for 48 h. In line with a previous report, compared with THP-1 cells transfected with the empty vector (NC), THP-1 cells with *SIRPB1* overexpression (*SIRPB1* ^*wt*^) exhibited increased activation of DAP12 and NF-κB and elicited tyrosine phosphorylation of Syk, Akt, and Jak2, causing increased IL-1β, TNF-α, and IL-6 release (Figures 5A,B,C,D,E). Furthermore, compared with *SIRPB1* ^*wt*^ cells, THP-1 cells expressing variant *SIRPB1* (*SIRPB1* ^*11143iG*^) displayed higher SIRPβ expression, enhanced activation of DAP12 and NF-κB, increased tyrosine phosphorylation of Syk, AKT, and Jak2, and higher secretion of IL-1β, TNF-α, and IL-6 (Figures 5A,B,C,D,E). Taken together, our results show that the frameshift variant of *SIRPB1* amplified the function of the wild-type *SIRPB1* gene.

## Discussion

SIRPs are a class of cell surface signaling receptors that are produced differently in myeloid and neural cells. They each include three extracellular Ig-like domains (Adams et al., 1998; Kharitonenkov et al., 1997). Despite having very identical extracellular domains, SIRPs can be distinguished as activating (α) or inhibitory (β) isoforms using conventional patterns in their cytoplasmic or transmembrane regions (Kharitonenkov et al., 1997). SIRPα, which binds to its ligand CD47, was found to inhibit signaling pathways that are mediated by receptor tyrosine kinase, as its cytoplasmic domains contain immunoreceptor tyrosine-based inhibitory motifs (ITIMs), which recruit the phosphatase SH2-domain-containing proteins SHP-1 and SHP-2 *in vivo* (Barclay and Brown, 2006). Contrary to SIRPα, SIRPβ lacks the sequence patterns necessary to attach SHP-1 and SHP-2 in its short six-amino acid cytoplasmic domain. The transmembrane domain of SIRPβ, however, has a charged amino acid residue that can bind to DAP12, which possesses a single cytoplasmic ITAM, and activate cell-mediated cytotoxicity and cytokine release (Dietrich et al., 2000; Liu et al., 2005; McVicar et al., 1998).

In the present study, a rare frameshift variant (c.1143_1144insG; p. Leu381_Leu382fs) in the innate immunity gene *SIRPB1* was identified. Genotyping analysis revealed that the novel frameshift variant in *SIRPB1* was significantly enriched in patients compared to that in healthy controls. Based on publicly available gene expression data, our research showed that patients with CD in the active stage had considerably higher relative expression of SIRPβ, indicating that SIRPβ is involved in the progression of intestinal inflammation. In addition, the frameshift variant of *SIRPB1* leads to an alteration of the amino acid sequence located both in the transmembrane and cytoplasmic regions. Therefore, we tested the functional contribution of the frameshift variant of *SIRPB1*. Our data show that, compared with WT controls, THP-1 cells transfected with the variant *SIRPB1* sequence displayed higher expression of SIRPβ and its adaptor protein DAP12, enhanced activation of subsequent signal transduction, and increased pro-inflammatory cytokines production and NF-κB expression. Accordingly, IHC data of ileocolonic tissue implied that patients with CD and variant *SIRPB1* expressed higher levels of SIRPβ and its adaptor protein DAP12. Collectively, we found a rare frameshift variant (c.1143_1144insG; p. Leu381_Leu382fs) in *SIRPB1* that leads to alterations in the amino acid sequence located both in the transmembrane and cytoplasmic domains, could contribute to inflammation exacerbation by promoting the expression of SIRPβ and its adaptor protein, DAP12. We hypothesized that the frameshift variant in *SIRPB1* may result in conformational changes of SIRPβ or form a functional cytoplasmic domain owing to a longer amino acid sequence, which needs to be elucidated in future studies.

In our study, the data analysis shows that the rare gain-of-function frameshift variant (c.1143_1144insG; p. Leu381_Leu382fs) in *SIRPB1* is associated with Han Chinese patients with CD and provides insights that the variant in *SIRPB1* upregulated the activation of NF-κB and increased the production of IL-1β, TNF-α, and IL-6 by inducing DAP12, Syk, Akt, and Jak2 to become tyrosine phosphorylated in macrophages, CD pathogenesis is facilitated. This study has several limitations. The familiar CD patients and the subsequent cohort for replication analyses are all Han Chinese from a single IBD center, so, further validation in other population is necessary. As well, due to limited budget and lack of suitable primers, only 61 variants were validated in replication analyses. Although the function of frameshift variant in SIRPB1 were explored *in vitro* using THP-1 cells, further investigation using other cell lines and also *in vivo* are required. And also, for replication analysis, a *p*-value <0.05 without multiple testing correction was used in validation, so, further function study was adopted to verify the susceptive possibility of the SIRPB1 variant in CD patients.

## Data Availability

The data presented in the study are deposited in the SRA repository and can be accessed at: https://dataview.ncbi.nlm.nih.gov/object/PRJNA962047.
