# Supplementary material for: A frameshift variant in the SIRPB1 gene confers susceptibility to Crohn’s disease in a Chinese population
Source: Front Genet. 2023 May 30;14:1130529. doi: 10.3389/fgene.2023.1130529 (PMC10267704; doi:10.3389/fgene.2023.1130529)
Supplement: Supplementary file 2 [file Table5.DOCX]

Supplementary_Table_5. the specimen sources of patients and health control for IHC

| Patient number | diagnosis | SIRPB1 mutation | Specimen location | specimen source |
| --- | --- | --- | --- | --- |
| 1 | CD | positive | ileocecal valve | endoscopic biopsy |
| 2 | CD | positive | terminal ileum; colon | endoscopic biopsy |
| 3 | CD | positive | ileocecal valve | endoscopic biopsy |
| 4 | CD | positive | terminal ileum | endoscopic biopsy |
| 5 | CD | positive | terminal ileum | endoscopic biopsy |
| 6 | CD | positive | terminal ileum | surgical specimen |
| 7 | CD | positive | terminal ileum | endoscopic biopsy |
| 8 | CD | positive | terminal ileum; colon | surgical specimen |
| 9-15 | CD | negative | terminal ileum | surgical specimen |
| 16-18 | Health control | negative | terminal ileum | endoscopic biopsy |
