# Supplementary material for: A frameshift variant in the SIRPB1 gene confers susceptibility to Crohn’s disease in a Chinese population
Source: Front Genet. 2023 May 30;14:1130529. doi: 10.3389/fgene.2023.1130529 (PMC10267704; doi:10.3389/fgene.2023.1130529)
Supplement: Supplementary file 4 [file Table3.DOCX]

**Supplementary_Table_3**  Candidate variants list generated from genome-wide association studies

| Gene | rs number | Unique ID | Mutation type | Mutation consequence | Gene-note |
| --- | --- | --- | --- | --- | --- |
| *PCNXL2* | [rs759917992](https://www.ncbi.nlm.nih.gov/snp/rs759917992) | chr1:233386580T>C | SNV | Splice acceptor |  |
| *TP73* | [rs1641123267](https://www.ncbi.nlm.nih.gov/snp/rs1641123267) | chr1:3638706C>T | SNV | Missense |  |
| *UBE2U* | [rs776567811](https://www.ncbi.nlm.nih.gov/snp/rs776567811) | chr1:64676467AC>A | Del | Frameshift |  |
| *LSP1* | NA | chr11:1891892G>A | SNV | Splice donor | Known IBD gene |
| *C11orf42* | [rs751350529](https://www.ncbi.nlm.nih.gov/snp/rs751350529) | chr11:6231170C>T | SNV | Stop gained |  |
| *C11orf42* | [rs749213397](https://www.ncbi.nlm.nih.gov/snp/rs749213397) | chr11:6231682G>A | SNV | Stop gained |  |
| *ALG8* | [rs1488580557](https://www.ncbi.nlm.nih.gov/snp/rs1488580557) | chr11:77813960G>A | SNV | Stop gained |  |
| *POLR3B* | [rs2036678883](https://www.ncbi.nlm.nih.gov/snp/rs2036678883) | chr12:106770153C>T | SNV | Missense |  |
| *OAS3* | [rs771052891](https://www.ncbi.nlm.nih.gov/snp/rs771052891) | chr12:113385843GC>G | Del | Frameshift |  |
| *MUC19* | [rs1411441474](https://www.ncbi.nlm.nih.gov/snp/rs1411441474) | chr12:40834995T>A | SNV | Missense | Known IBD gene |
| *MYO1A* | NA | chr12:57440644TAC>T | Del | Splice donor |  |
| *A2ML1* | [rs766100204](https://www.ncbi.nlm.nih.gov/snp/rs766100204) | chr12:8998099G>A | SNV | Splice donor |  |
| *EPSTI1* | NA | chr13:43537471T>TA | Ins | Frameshift |  |
| *DAAM1* | NA | chr14:59782026G>T | SNV | Missense |  |
| *SYNE2* | [rs777169796](https://www.ncbi.nlm.nih.gov/snp/rs777169796) | chr14:64519635A>T | SNV | Stop gained |  |
| *CPPED1* | [rs748886359](https://www.ncbi.nlm.nih.gov/snp/rs748886359) | chr16:12798820C>T | SNV | Missense |  |
| *BEAN1* | [rs989514270](https://www.ncbi.nlm.nih.gov/snp/rs989514270) | chr16:66471600C>A | SNV | Stop gained |  |
| *AATF* | [rs1306922955](https://www.ncbi.nlm.nih.gov/snp/rs1306922955) | chr17:35307665G>A | SNV | Stop gained |  |
| *ZPBP2* | [rs1460554471](https://www.ncbi.nlm.nih.gov/snp/rs1460554471) | chr17:38024800A>G | SNV | Splice acceptor | Known IBD gene |
| *PDK2* | [rs748085033](https://www.ncbi.nlm.nih.gov/snp/rs748085033) | chr17:48185985C>T | SNV | Stop gained |  |
| *ENO3* | [rs764120380](https://www.ncbi.nlm.nih.gov/snp/rs764120380) | chr17:4856098C>T | SNV | Stop gained |  |
| *EPX* | [rs757233476](https://www.ncbi.nlm.nih.gov/snp/rs757233476) | chr17:56281773C>T | SNV | Stop gained |  |
| *COL5A3* | NA | chr19:10079057  TCACAGGGTCTCC>T | Del | Splice donor |  |
| *TYK2* | NA | chr19:10479075  GAAGC>G | Del | Frameshift |  |
| *GDF1* | [rs1568291627](https://www.ncbi.nlm.nih.gov/snp/rs1568291627) | chr19:18981025  ACGGGGGCG>A | Del | Frameshift |  |
| *C19orf40* | [rs760353712](https://www.ncbi.nlm.nih.gov/snp/rs760353712) | chr19:33464372C>CTT | Ins | Frameshift |  |
| *VRK3* | [rs1048569809](https://www.ncbi.nlm.nih.gov/snp/rs1048569809) | chr19:50528523C>G | SNV | Splice donor |  |
| *DFNB59* | [rs1437628682](https://www.ncbi.nlm.nih.gov/snp/rs1437628682) | chr2:179320735A>G | SNV | Splice acceptor |  |
| *VRK2* | [rs1328945383](https://www.ncbi.nlm.nih.gov/snp/rs1328945383) | chr2:58312086G>A | SNV | Splice donor |  |
| *WDPCP* | NA | chr2:63486522T>TC | Ins | Frameshift |  |
| *ANKEF1* | [rs752349062](https://www.ncbi.nlm.nih.gov/snp/rs752349062) | chr20:10019057C>A | SNV | Stop gained |  |
| *SIRPB1* | [rs1275744950](https://www.ncbi.nlm.nih.gov/snp/rs1275744950) | chr20:1546854G>GC | Ins | Frameshift |  |
| *CPNE1* | NA | chr20:34214629C>CT | Ins | Frameshift |  |
| *PLCG1* | NA | chr20:39792446A>T | SNV | Missense |  |
| *FBXO40* | NA | chr3:121341344CT>C | Del | Frameshift |  |
| *ERC2* | [rs1293707325](https://www.ncbi.nlm.nih.gov/snp/rs1293707325) | chr3:56183136G>A | SNV | Stop gained |  |
| *MMAA* | [rs757548934](https://www.ncbi.nlm.nih.gov/snp/rs757548934) | chr4:146572222C>T | SNV | Stop gained |  |
| *KLHL5* | [rs755006031](https://www.ncbi.nlm.nih.gov/snp/rs755006031) | chr4:39105132G>GT | Indel | Splice donor |  |
| *SEC31A* | NA | chr4:83788384G>A | SNV | Missense |  |
| *SLC2A9* | NA | chr4:9922067C>T | SNV | Stop gained |  |
| *FBXL21* | [rs201662172](https://www.ncbi.nlm.nih.gov/snp/rs201662172) | chr5:135273232C>A | SNV | Stop gained |  |
| *PCDH12* | NA | chr5:141336148C>T | SNV | Stop gained |  |
| *GHR* | rs752025877 | chr5:42565977A>G | SNV | Start lost |  |
| *SYNJ2* | NA | chr6:158438246  AAAGG>A | Del | Frameshift |  |
| *CAGE1* | [rs1414911763](https://www.ncbi.nlm.nih.gov/snp/rs1414911763) | chr6:7329418G>A | SNV | Stop gained |  |
| *SH2B2* | NA | chr7:101960938C>T | SNV | Stop gained |  |
| *RPA3* | rs529874466 | chr7:7758145C>G | SNV | Splice donor |  |
| *CD36* | rs748202229 | chr7:80285946C>T | SNV | Stop gained |  |
| *TNFSF15* | NA | chr9:117568285T>C | SNV | Missense | Known IBD gene |
| *LRSAM1* | rs770106776 | chr9:130265135G>A | SNV | Missense |  |
| *HMCN2* | rs1445146226 | chr9:133245203G>A | SNV | Stop gained |  |
| *SDCCAG3* | rs375609278 | chr9:139301649C>G | SNV | Missense variant | Known IBD gene |
| *PRSS3* | NA | chr9:33794797TGA>T | Del | Frameshift |  |
| *CCL27* | rs746707552 | chr9:34662369G>A | SNV | Stop gained |  |
| *PCSK5* | rs769457551 | chr9:78973443C>T | SNV | Stop gained |  |
| *NOD2* | rs104895438 | chr16:50745656G>A | SNV | Missense | Known IBD gene |
| *MUC19* | rs112524759 | chr12:40882387TA>T | Del | Frameshift | Known IBD gene |
| *BIRC8* | rs145690856 | chr19:53793456G>A | SNV | Stop gained |  |
| *XPA* | rs149226993 | chr9:100447247G>A | SNV | Stop gained |  |
| *GLB1* | rs192732174 | chr3:33109737G>A | SNV | Missense |  |
| *ERCC4* | rs2020959 | chr16:14041622C>A | SNV | Stop gained |  |

NA: Not available.
