# Supplementary material for: A frameshift variant in the SIRPB1 gene confers susceptibility to Crohn’s disease in a Chinese population
Source: Front Genet. 2023 May 30;14:1130529. doi: 10.3389/fgene.2023.1130529 (PMC10267704; doi:10.3389/fgene.2023.1130529)
Supplement: Supplementary file 6 [file Table6.DOCX]

**Supplementary_Table_6** The cloned sequences of SIRPB1 gene for plasmid transfection.

**WT Sequence**

<https://www.ncbi.nlm.nih.gov/nuccore/NM_006065.4/>

>NM_006065.4:151-1347 Homo sapiens signal regulatory protein beta 1 (SIRPB1), transcript variant 1, mRNA

ATGCCCGTGCCAGCCTCCTGGCCCCACCTTCCTAGTCCTTTCCTGCTGATGACGCTACTGCTGGGGAGACTCACAGGAGTGGCAGGTGAGGACGAGCTACAGGTGATTCAGCCTGAAAAGTCCGTATCAGTTGCAGCTGGAGAGTCGGCCACTCTGCGCTGTGCTATGACGTCCCTGATCCCTGTGGGGCCCATCATGTGGTTTAGAGGAGCTGGAGCAGGCCGGGAATTAATCTACAATCAGAAAGAAGGCCACTTCCCACGGGTAACAACTGTTTCAGAACTCACAAAGAGAAACAACCTGGACTTTTCCATCAGCATCAGTAACATCACCCCAGCAGACGCCGGCACCTACTACTGTGTGAAGTTCCGGAAAGGGAGCCCTGACGACGTGGAGTTTAAGTCTGGAGCAGGCACTGAGCTGTCTGTGCGCGCCAAACCCTCTGCCCCCGTGGTATCGGGCCCTGCGGTGAGGGCCACACCTGAGCACACAGTGAGCTTCACCTGCGAGTCCCATGGCTTCTCTCCCAGAGACATCACCCTGAAATGGTTCAAAAATGGGAATGAGCTCTCAGACTTCCAGACCAACGTGGACCCCGCAGGAGACAGTGTGTCCTACAGCATCCACAGCACAGCCAGGGTGGTGCTGACCCGTGGGGACGTTCACTCTCAAGTCATCTGCGAGATAGCCCACATCACCTTGCAGGGGGACCCTCTTCGTGGGACTGCCAACTTGTCTGAGGCCATCCGAGTTCCACCCACCTTGGAGGTTACTCAACAGCCCATGAGGGCAGAGAACCAGGCAAACGTCACCTGCCAGGTGAGCAATTTCTACCCCCGGGGACTACAGCTGACCTGGTTGGAGAATGGAAATGTGTCCCGGACAGAAACAGCTTCGACCCTCATAGAGAACAAGGATGGCACCTACAACTGGATGAGCTGGCTCCTGGTGAACACCTGTGCCCACAGGGACGATGTGGTGCTCACCTGTCAGGTGGAGCATGATGGGCAGCAAGCAGTCAGCAAAAGCTATGCCCTGGAGATCTCAGCGCACCAGAAGGAGCACGGCTCAGATATCACCCATGAAGCAGCGCTGGCTCCTACTGCTCCACTCCTCGTAGCTCTCCTCCTGGGCCCCAAGCTGCTACTGGTGGTTGGTGTCTCTGCCATCTACATCTGCTGGAAACAGAAGGCCTGA

**Mutant sequence**

ATGCCCGTGCCAGCCTCCTGGCCCCACCTTCCTAGTCCTTTCCTGCTGATGACGCTACTGCTGGGGAGACTCACAGGAGTGGCAGGTGAGGACGAGCTACAGGTGATTCAGCCTGAAAAGTCCGTATCAGTTGCAGCTGGAGAGTCGGCCACTCTGCGCTGTGCTATGACGTCCCTGATCCCTGTGGGGCCCATCATGTGGTTTAGAGGAGCTGGAGCAGGCCGGGAATTAATCTACAATCAGAAAGAAGGCCACTTCCCACGGGTAACAACTGTTTCAGAACTCACAAAGAGAAACAACCTGGACTTTTCCATCAGCATCAGTAACATCACCCCAGCAGACGCCGGCACCTACTACTGTGTGAAGTTCCGGAAAGGGAGCCCTGACGACGTGGAGTTTAAGTCTGGAGCAGGCACTGAGCTGTCTGTGCGCGCCAAACCCTCTGCCCCCGTGGTATCGGGCCCTGCGGTGAGGGCCACACCTGAGCACACAGTGAGCTTCACCTGCGAGTCCCATGGCTTCTCTCCCAGAGACATCACCCTGAAATGGTTCAAAAATGGGAATGAGCTCTCAGACTTCCAGACCAACGTGGACCCCGCAGGAGACAGTGTGTCCTACAGCATCCACAGCACAGCCAGGGTGGTGCTGACCCGTGGGGACGTTCACTCTCAAGTCATCTGCGAGATAGCCCACATCACCTTGCAGGGGGACCCTCTTCGTGGGACTGCCAACTTGTCTGAGGCCATCCGAGTTCCACCCACCTTGGAGGTTACTCAACAGCCCATGAGGGCAGAGAACCAGGCAAACGTCACCTGCCAGGTGAGCAATTTCTACCCCCGGGGACTACAGCTGACCTGGTTGGAGAATGGAAATGTGTCCCGGACAGAAACAGCTTCGACCCTCATAGAGAACAAGGATGGCACCTACAACTGGATGAGCTGGCTCCTGGTGAACACCTGTGCCCACAGGGACGATGTGGTGCTCACCTGTCAGGTGGAGCATGATGGGCAGCAAGCAGTCAGCAAAAGCTATGCCCTGGAGATCTCAGCGCACCAGAAGGAGCACGGCTCAGATATCACCCATGAAGCAGCGCTGGCTCCTACTGCTCCACTCCTCGTAGCTCTCCTCCTGGGCCCCAAGCTGcCTACTGGTGGTTGGTGTCTCTGCCATCTACATCTGCTGGAAACAGAAGGCCTGACTGACCCTCAGTCTCTGCTGCCTCCTCCTTTCTTGAGAAGCTCAGCCTGA
